# Viral-bacterial co-infection in Australian Indigenous children with acute otitis media

**DOI:** 10.1186/1471-2334-11-161

**Published:** 2011-06-07

**Authors:** Michael J Binks, Allen C Cheng, Heidi Smith-Vaughan, Theo Sloots, Michael Nissen, David Whiley, Joseph McDonnell, Amanda J Leach

**Affiliations:** 1 Ear and Respiratory Unit, Child Health Division, Menzies School of Health Research, Charles Darwin University, Darwin 0810, Australia; 2 Department of Epidemiology and Preventive Medicine, Faculty of Medicine, Monash University, Melbourne 3800, Australia; 3 Queensland Paediatric Infectious Diseases Laboratory, Sir Albert Sakzewski Virus Research Centre, Royal Children's Hospital, Brisbane 4029, Australia

## Abstract

**Background:**

Acute otitis media with perforation (AOMwiP) affects 40% of remote Indigenous children during the first 18 months of life. *Streptococcus pneumoniae*, *Haemophilus influenzae *and *Moraxella catarrhalis *are the primary bacterial pathogens of otitis media and their loads predict clinical ear state. Our hypothesis is that antecedent respiratory viral infection increases bacterial density and progression to perforation.

**Methods:**

A total of 366 nasopharyngeal swabs from 114 Indigenous children were retrospectively examined. A panel of 17 respiratory viruses was screened by PCR, and densities of *S. pneumoniae, H. influenzae *and *M. catarrhalis *were estimated by quantitative real time PCR. Data are reported by clinical ear state.

**Results:**

*M. catarrhalis *(96%), *H. influenzae *(91%), *S. pneumoniae *(89%) and respiratory viruses (59%) were common; including rhinovirus (HRV) (38%), polyomavirus (HPyV) (14%), adenovirus (HAdV) (13%), bocavirus (HBoV) (8%) and coronavirus (HCoV) (4%). Geometric mean bacterial loads were significantly higher in children with acute otitis media (AOM) compared to children without evidence of otitis media. Children infected with HAdV were 3 times more likely (p < 0.001) to have AOM with or without perforation.

**Conclusion:**

This study confirms a positive association between nasopharyngeal bacterial load and clinical ear state, exacerbated by respiratory viruses, in Indigenous children. HAdV was independently associated with acute ear states.

## Background

Australian Aboriginal children have the highest published prevalence of acute otitis media (AOM), with and without perforation, in the world [1]. Infants are colonized with multiple species of respiratory bacteria within weeks of birth, including the AOM pathogens, *S. pneumoniae*, *H. influenzae *and *M. catarrhalis*. Early dense colonization predicts early onset of otitis media, and progression to tympanic membrane perforation (TMP) occurs in 30% of children by 6 months of age and 40% by 18 months of age [2,3]. At any one time few as 8% of remote Indigenous children aged 6 - 30 months have bilaterally normal middle ears [1].

Understanding the burden of otitis media (OM) in remote indigenous populations is complex. Overcrowding [4], poor hygiene [5], limited formal education [6], and inadequate access to under-resourced medical services contribute to the prevalence and severity of OM in these children [2,3]. Recurrent OM and perforation is associated with conductive hearing loss and further contributes to poor educational outcomes [7].

To date, interventions for otitis media have had modest benefits for Indigenous children in remote communities [8]. A randomised controlled trial in the Northern Territory, comparing antibiotics for the treatment of AOM, showed no difference in the risk of clinical failure between amoxicillin (54% failure rate) and azithromycin (50% failure rate) treatment groups despite significant reductions in carriage density of *S. pneumoniae *and *H. influenzae *in the azithromycin group [9].

The introduction of the seven-valent pneumococcal vaccine (7vPCV) in 2001 brought anticipation of a reduced OM burden. However a review of randomised controlled trials using PCVs to prevent AOM in children aged 12 years or younger, sourced from the Cochrane Central Register of Controlled Trials, found that the vaccine administered during infancy induced only a marginal reduction in OM disease (6-7%) [10]. In Australia a birth cohort study of Indigenous infants found a limited benefit of 7vPCV on OM compared to historic controls. No effect was observed on the proportion of children with otitis media with effusion (OME), AOM or AOMwiP at 12 months of age, however, significantly fewer vaccinees (14%) than controls (23%) experienced chronic suppurative otitis media (CSOM) and vaccinees experienced delayed onset of TMP [11].

A plausible explanation for the poor response to antibiotics and high prevalence of AOM with perforation in the Indigenous community setting is co-infection of multiple bacterial pathogens with respiratory viruses. Human rhinovirus (HRV), respiratory syncytial virus (RSV), influenza virus A and B (FLUA, FLUB), HAdV, HCoV and parainfluenza virus 1, 2 and 3 (PIV 1, PIV2 & PIV3) are commonly associated with OM [12-14]. Recent advances in molecular viral diagnostics [15] now allow a broader insight into viral OM pathogenesis.

Infection with respiratory viruses induces damage to the epithelial mucosa of airways, Eustachian tube and middle ear exposing surface elements that bacteria can adhere to [16]. The interaction between FLUA and *S. pneumoniae *is well noted [17,18]. Influenza neuraminidase is demonstrated to increase pneumococcal adhesion and invasion in the Eustachian tube and middle ear [19,20]. RSV can also facilitate bacterial adhesion. Studies reveal that in the presence of RSV, certain strains of *M. catarrhalis *have an enhanced ability to adhere to epithelial cells [21]. Additionally, viral mediated inflammation congests the Eustachian tube and further reduces cilial function, inhibiting bacterial clearance [22,23], while viral immunosuppression can lead to bacterial superinfection [24]. Recent evidence suggests that pneumococcal load is driven by FLUA, HRV and RSV infection, and that enhanced transmission and acquisition occurs in the presence of FLUA [18,25].

For young Indigenous children, the role of respiratory viruses in OM remains unclear. In the tropical North of Australia, AOM is endemic in remote communities and not subject to seasonal viral outbreaks. In this study we use molecular methods [3,15] to investigate whether antecedent nasopharyngeal viral infection increases bacterial load and progression to AOMwiP.

## Methods

### Population and primary data

Samples were sourced from two previous studies based in the same remote Aboriginal communities. The first, the COMIT1 trial (1996-2001), was a birth cohort randomized controlled trial that examined the effect of long course amoxicillin versus placebo on resolution of first detected OME and the prevention of tympanic membrane perforation [8]. The second, the PRIORiTI (PRevenar Immunisation for Otitis media Reductions in Tiwi Infants) study (2001-2004), was a birth cohort observational study following the introduction of routine childhood heptavalent conjugate pneumococcal vaccination (7vPCV) [11]. In both studies examinations were undertaken at approximately one month intervals from birth till 24 months.

Sample collection and clinical definitions have been described previously [8,11]. In summary, ear state was defined as follows: (a) Normal: normal or minor pathology (abnormal appearance or retracted drum), (b) OME: either a normal tympanic membrane (TM) or intact, non-bulging TM, plus type B tympanogram, (c) AOM: bulging TM, without perforation, and type B tympanogram, (d) AOMwiP: middle ear discharge present for less than 6 weeks and perforation covering less than 2% of the pars tensa of the TM. (e) CSOM: middle ear discharge present for longer than 6 weeks and perforation covering greater than 2% of the pars tensa of the TM. The final middle ear diagnosis reflects the child's most severely affected ear. As the focus of this study was acute ear pathology, swabs from children with CSOM were excluded.

Analysis of bacterial and viral pathogens was performed as part of a larger case-control/crossover study (Cheng et al, manuscript in preparation). Bacterial and viral DNA was extracted by defined methods [3,15,26,27] and quantitative real time polymerase chain reaction (RTQ-PCR) was used to determine total and species specific pathogen loads[3]. The PCR target genes were: 16S rRNA for total bacterial load [28], autolysin (*lytA*) for *S. pneumoniae *load [29], protein D (*hpd*) for *H. influenzae *load [3], and the outer membrane protein (*copB*) for *M. catarrhalis *load [30]. Specimens were tested for FLUA and B, RSV, PIV 1, 2 and 3, HAdV, human meta-pneumovirus (HMPV), HRV, enterovirus (EV), HCoV's (HKU1, OC43, 229E and NL63), HBoV and HPyV's (WU and KI) by PCR as previously described [15,26,27].

### Ethics

Informed written consent was obtained from the parents of all children who participated in the parent studies, COMIT and PRIORiTI. This work was performed following consultation with Indigenous community members, and was approved by the Human Research Ethics Committee of the Menzies School of Health Research and the Department of Human Services (HREC - 07/78).

### Statistical Analysis

Chi-square or Fischer's exact analysis was used to compare the proportions of virus and bacteria positive swabs in each of the four ear states. Bacterial loads were log normally distributed and therefore were log transformed for analysis. Non-parametric tests for continuous variables, Kruskal-Wallis equality of populations rank test and Mann Whitney Wilcoxon ranksum tests, were used to compare bacterial loads between ear states, with or without viral co-detection. Linear regression was used to examine the relationships between the bacterial loads and interacting variables such as age and antibiotic use (<2 weeks before swab was taken) with lower limits set at lowest positive load value. The reported p values are two-tailed and significance was set at 5%. All statistical analysis was performed using STATA IC version 11 (StataCorp, Texas, USA).

## Results

### Bacterial and viral infection of the nasopharynx in relation to ear state

Of the 366 swabs from 114 children, 58 (16%) swabs were from children diagnosed with tympanic membrane perforation. 115 (31%) had AOM without perforation, 175 (48%) had OME, and 18 (5%) were from children with Normal ears (Table 1).

**Table 1:** Data description by clinical ear state

*Variable*	Total	Normal	OME	AOM	AOMwiP	*P*	
Number of kids	114	-	-	-	-	na	
Number of swabs (% of total)	366	18(5)	175(48)	115(31)	58(16)	na	
Mean age months (median)	8.0	5.4 (3.4)	7.2 (6.6)	8. (7.7)	8.5 (8.1)	**<0.001**	*

**Bacteria detection (% of total)**							

Any bacteria	366(100)	18(100)	175(100)	115(100)	58(100)	na	**
Spn	327(89)	11(61)	155(89)	109(95)	52(90)	**<0.001**	**
Hinf	333(91)	16(89)	155(89)	110(96)	52(90)	0.211	**
Mcat	351(96)	14(78)	167(95)	113(98)	57(98)	**<0.001**	**

**Virus detection (% of total)**							

Any virus	228(62)	12(67)	102(58)	78(68)	36(62)	0.416	**
Total virus (virus:swab(+)ratio)	312(1.37)	14(1.17)	134(1.31)	117(1.51)	47(1.31)	0.120	**
HBoV	28(8)	0(0)	14(8)	10(9)	4(7)	0.627	**
HPyV	50(14)	6(33)	20(11)	19(17)	5(9)	**0.034**	**
HAdV	49(13)	1(6)	13(7)	22(19)	13(22)	**0.003**	**
HRV	139(38)	5(28)	69(39)	47(41)	18(31)	0.467	**
HCoV	14(4)	0(0)	7(4)	5(4)	2(3)	0.840	**
Other	32(9)	2(11)	11(6)	14(12)	5(9)	0.369	**

*P *shows the significance of the values across all ear states using either the: (*) Kruskal-Wallis equality-of-populations rank test, or the (**) Chi-square test.

By RTQ-PCR most swabs were positive for *M. catarrhalis *(96%), *H. influenzae *(91%) and *S. pneumoniae *(89%). Greater than 80% had all three and all had at least one of these pathogens. Although the proportion of positive swabs was high for all ear states, more children from the AOM group were positive for each of the bacteria compared to the Normal group (Table 1).

Sixty two percent (228/366) of swabs amplified at least one virus. A total of 312 viruses were detected in the 228 positive swabs at an average of 1.37 viruses per positive swab. HRV (38%) was the most frequently encountered virus, followed by HPyV (14%), HAdV (13%), HBoV (8%) and HCoV (4%). Other viruses (FLUA and B, PIV 1,2 and 3, HMPV, RSV and EV) were only found in <9% collectively (Table 1). Further investigation of the HPyV subtypes, WU and KI, revealed the WU virus predominated (92% of HPyV positive swabs).

**Figure 1:**
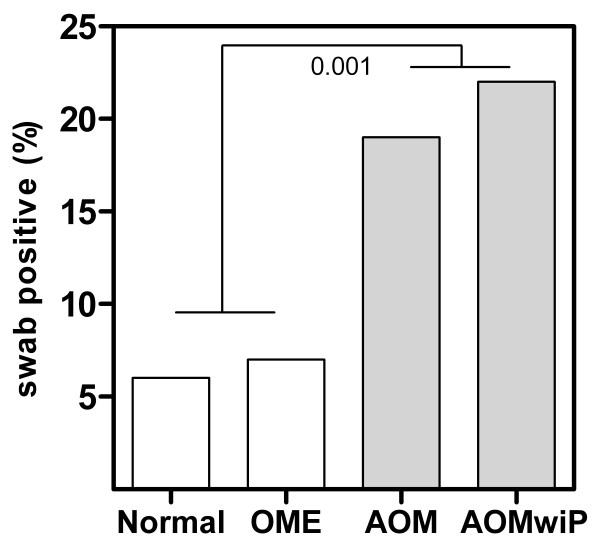
**Proportion of HAdV positive nasopharyngeal swabs by clinical ear state**. A significantly greater number of swabs were HAdV positive in children with acute OM with and without perforation.

The proportions of HAdV detected in the nasopharyngeal swabs for each ear diagnosis were: 6% for Normal, 7% for OME, 19% for AOM and 22% for AOMwiP (Table 1) (Figure 1). No other virus revealed a positive relationship with ear state.

### Bacterial load in the nasopharynx in relation to ear state

**Figure 2:**
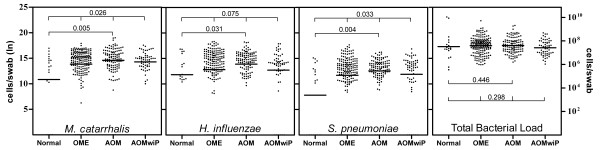
**Bacterial load versus Clinical Ear State**. The geometric mean bacterial loads are segregated by clinical ear state for each bacterial pathogen. A significantly higher load was seen for *M. catarrhalis, H. influenzae *and *S. pneumoniae *in children with AOM compared to Normal ear states (Mann Whitney Wilcoxon test). Significant differences in the loads across the ear states, for *M. catarrhalis and S. pneumoniae*, were confirmed by the Kruskal-Wallis equality of populations rank test. There was no significant difference in geometric mean of the Total Bacterial Load between ear states.

The geometric-mean bacterial loads were high throughout. Individual bacterial loads showed stepwise elevations by categorical ear state for *M. catarrhalis, H. influenzae *and *S. pneumoniae*, peaking at AOM (Figure 2). Unexpectedly, the individual bacterial loads were lower at the time of AOM with perforation than at AOM without perforation. A dichotomous ranksum test revealed a significantly higher load in the AOM group compared to Normal group for *M. catarrhalis *(N = 5.42 × 10^4^, AOM = 2.19 × 10^6^; p = 0.005); *H. influenzae *(N = 1.33 × 10^5^, AOM = 1.09 × 10^6^; p = 0.031); and *S. pneumoniae *(N = 2.44 × 10^3^, AOM = 2.68 × 10^5^; p = 0.004). The total bacterial loads were all greater than 10^7^, showing no trend for ear state.

### Bacterial load in the nasopharynx when viruses were present

**Figure 3:**
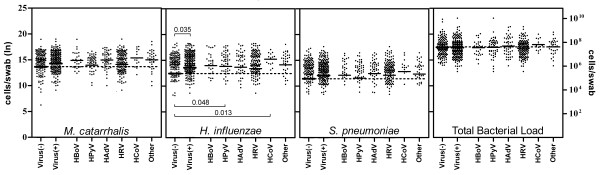
**Bacterial Loads in the presence of common respiratory viruses**. Significantly higher load was found for *H. influenzae *in the presence of any study virus, HPyV and HCoV, compared to no viral co-infection. The dotted line represents the baseline mean load in the absence of viral co-infection.

In the presence of viruses the individual loads had a general trend upwards (Figure 3). The combined load of *M. catarrhalis, H. influenzae and S. pneumoniae *was greater when examined by presence or absence of any study virus (p = 0.035) (data not shown). Of the individual bacterial pathogens, significantly higher loads were only seen for *H. influenzae *co-infected with any study virus (Virus +), HPyV and HCoV (Table 2) (Figure 3). Despite the association between HAdV and ear disease, load increases in the presence of adenovirus were insignificant.

**Table 2:** Univariate linear regression analysis of bacterial load by known variables

	Variables		N	Geometric Mean Bacterial Load (SD)	Coefficient (95% CI)	*P*
***M. catarrhalis***	**Sex**	Male	180	14.26(2.89)	-0.29(-0.82 to 0.24)	0.279
		Female	186	13.77(3.89)		
	**Age category**	<3mo	36	11.59(5.96)	-2.31(-3.44 to -1.18)	**<0.001**
		3-6mo	95	14.41(2.92)	-0.45(-1.37 to 0.47)	0.336
		6-9mo	122	13.85(3.51)	-0.88(-1.77 to 0.00)	0.051
		9-12mo	71	14.50(1.77)	-0.47(-1.44 to -0.49)	0.335
		>12mo	42	14.84(2.68)	Baselevel	--
		Overall				**0.001**
	**Antibiotics**	No	193	13.78(3.57)	0.42(-0.11 to 0.95)	0.123
	**(<2 weeks)**	Yes	173	14.27(3.29)		
	**Number of viruses**	0	138	13.51(4.09)	Baselevel	--
		1	153	14.08(3.42)	0.42(-0.17 to 1.01)	0.166
		>1	75	14.79(1.56)	0.92(0.20 to 1.64)	**0.013**
		Overall				**0.042**

***H. influenzae***	**Sex**	Male	180	12.96(4.61)	0.21(-0.38 to 0.80)	0.478
		Female	186	13.24(4.48)		
	**Age category**	<3mo	36	10.26(7.10)	-1.71(-3.0 to -0.43)	**0.009**
		3-6mo	95	13.46(4.50)	-0.01(-1.04 to 1.01)	0.981
		6-9mo	122	13.23(4.29)	-0.33(-1.32 to 0.66)	0.513
		9-12mo	71	13.36(3.71)	-0.32(-1.39 to 0.76)	0.565
		>12mo	42	13.91(2.80)	Baselevel	--
		Overall				**0.041**
	**Antibiotics**	Yes	193	12.82(4.96)	0.22(-0.36 to 0.81)	0.455
	**(<2 weeks)**	No	173	13.41(4.00)		
	**Number of viruses**	0	138	12.35(5.13)	Baselevel	--
		1	153	13.34(4.35)	0.65(-0.01 to 1.30)	**0.05**
		>1	75	13.98(3.46)	1.00(0.21 to 1.81)	**0.013**
		Overall				**0.029**

***S. pneumoniae***	**Sex**	Male	180	12.22(3.96)	-0.41(-0.93 to 0.11)	0.119
		Female	186	11.39(4.80)		
	**Age category**	<3mo	36	9.83(6.03)	-1.13(-2.26 to -0.02)	**0.05**
		3-6mo	95	12.61(3.40)	0.22(-0.68 to 1.13)	0.627
		6-9mo	122	11.54(4.92)	-0.20(-1.08 to 0.67)	0.647
		9-12mo	71	11.89(3.84)	-0.37(-1.32 to 0.58)	0.448
		>12mo	42	12.25(3.82)	Baselevel	--
		Overall				0.083
	**Antibiotics**	Yes	193	11.84(4.25)	0.05(-0.47 to 0.56)	0.862
	**(<2 weeks)**	No	173	11.76(4.62)		
	**Number of viruses**	0	138	11.30(4.96)	Baselevel	--
		1	153	12.13(3.99)	0.34(-0.24 to 0.92)	0.252
		>1	75	12.04(4.19)	0.73(-0.35 to 1.07)	0.319
		Overall				0.444

**Total Bacterial Load**	**Sex**	Male	180	17.46(1.65)	-0.06(-0.40 to 0.28)	0.74
		Female	186	17.40(1.65)		
	**Age category**	<3mo	36	17.52(2.12)	0.27(-0.46 to 1.00)	0.464
		3-6mo	95	17.71(1.44)	0.46(-0.14 to 1.06)	0.131
		6-9mo	122	17.45(1.76)	0.21(-0.37 to 0.79)	0.48
		9-12mo	71	17.10(1.50)	-0.15(-0.78 to 0.48)	0.646
		>12mo	42	17.25(1.46)	Baselevel	--
		Overall				0.185
	**Antibiotics**	Yes	193	17.36(1.61)	0.14(-0.20 to 0.48)	0.414
	**(<2 weeks)**	No	173	17.51(1.69)		
	**Number of viruses**	0	138	17.42(1.68)	Baselevel	--
		1	153	17.42(1.70)	0.01(-0.37 to 0.39)	0.976
		>1	75	17.46(1.48)	0.03(-0.44 to 0.49)	0.901
		Overall				0.992

Antibiotic use included any systemic antimicrobial prescription for respiratory infection in the 2 weeks prior to examination and swabbing.

### Association between bacterial load and other known factors

The impact of sex, age, antibiotic use and number of viruses detected were assessed using linear regression (Table 2). The loads of *M. catarrhalis*, *H. influenzae *and *S. pneumoniae *were significantly lower in children less than 3 months of age. This was in part due to the greater proportion of children without detectable OM bacteria at this early age. In children older than 3 months the effect of age on load was neutral. Linear regression also revealed that the density of *M. catarrhalis *and *H. influenzae *was positively influenced by respiratory viral infection while the impact of sex and antibiotic use (<2 weeks before swab was taken) on load was negligible. Furthermore, comparison of total and pathogen specific loads with and without antibiotics, for each ear state, did not reveal any significant differences (data not included).

### Correlation between molecular bacterial load and semi-quantitative microbiology

The positive correlation between the bacterial loads determined by RTQ-PCR and clinical ear state was consistent with original semi-quantitative microbiology (data not included). Sensitivity was enhanced using RTQ-PCR. Of the 356 swabs cultured for *M. catarrhalis*, 82% were culture positive and 96% were RTQ-PCR positive. Similarly for *H. influenzae*; 75% were culture positive and 91% were RTQ-PCR positive. All 366 swabs were cultured for *S. pneumoniae *of which 68% were culture positive and 89% were RTQ-PCR positive. 14%, 20% and 23% of swabs were culture negative and RTQ-PCR positive for *M. catarrhalis*, *H. influenzae *and *S. pneumoniae *respectively. 1%, 5% and 2% of swabs were culture positive but RTQ-PCR negative respectively.

### Limitations of the study

The bacterial load estimates have limitations: The quantity of material obtained on the swab was assumed to be equal for all swabs; DNA extraction efficiency was considered to be 100%; and molecular weight calculations were based on estimates of GC content. Additionally when quantifying the total bacterial load using 16S primers, we used *S. pneumoniae *with 4 ribosomal operons as our control. The actual number of ribosomal operons varies greatly between species. Importantly, load values remain relative to each other throughout. It is worth noting that the *hpd *(protein D) target is likely to be present in closely related *Haemophilus *species including non-haemolytic *H. haemolyticus *and as such this study may over represent classical non-typeable *H. influenzae*.

Our swabs were collected at monthly intervals, so we have assumed that microbiology at the time of diagnosis best approximates the aetiology of that event. However our definition of the primary perforation included perforation of less than 6 weeks duration. The exact date of onset was not available. This analysis describes the nasal microbiology and ear status at the time of swab collection and presents the data for Normal, OME, AOM and AOMwiP. Furthermore, while the nasopharynx and the middle ear show concordance of infection, studies suggest a lapse of 3-10 days between upper respiratory symptoms and onset of otitis media [31,32]. It is therefore plausible that bacterial load and viral detection will be greater in swabs collected prior to perforation, compared to swabs collected at or after the time of perforation.

## Discussion

In this study the high bacterial carriage rates, high bacterial loads, and small number of children with Normal ears (5% of swabs), reflect the large burden of respiratory infection in Indigenous children who live in remote communities. Increases in individual respiratory bacterial load (and carriage) were apparent with worsening ear state with a peak at AOM, whereas the total bacterial load was high regardless of ear state.

The broad viral panel scrutinized by sensitive PCR methods in this study revealed a high proportion of virus positive nasopharyngeal swabs (62% swabs or 89% children). The proportions remained stable by ear state including the Normal group, of which 67% were positive for any study virus. HRVs, which are responsible for more cold-like illnesses than any other virus [33], were the most prevalent viruses identified in this study (38%), though no clear correlation between HRV and clinical ear state was observed.

HAdV was significantly associated with acute ear pathology (Figure 1). Children with AOM or AOMwiP were infected with HAdV at 3 times the rate of children with Normal and OME diagnosis (P < 0.001) though this was not associated with a significant increase in bacterial load. HAdV is increasingly reported as an upper respiratory pathogen with studies showing occurrence of AOM in up to 50% of those children infected [12-14,34]. No licensed HAdV treatment is currently available [35].

Overall our data supports a potential role for viruses in AOM, particularly HAdV, encouraging prospective studies and clinical trials to better understand the role viruses might have in endemic OM.

Differences in bacterial load with viral co-infection were apparent but of limited significance. *H. influenzae *load was significantly elevated in the presence of HPyV, HCoV and any study virus, and linear regression revealed that the loads of *M.catarrhalis *and *H. influenzae *were higher with detection of multiple viruses (Figure 3) (Table 2). At no point were the individual bacterial loads lower in the presence of a virus. We suspect that the consistently high bacterial loads seen in these children might mask the true effect of respiratory viruses on bacterial density.

The higher individual loads seen in AOM swabs compared to AOMwiP swabs were unexpected. We suggest that this is likely due to the mismatch in timing of onset and swab collection.

Regression modelling of bacterial load by age revealed significantly lower loads for all three individual bacterial pathogens in children less than 3 months of age. In children older than 3 months, age exerted no influence on load. This finding reflects the natural acquisition of these bacteria after birth. No differences in the bacterial loads were observed between the sexes or with antibiotic use in the fortnight prior to examination and swabbing (Table 2). Treatment guidelines recommend antibiotic use upon diagnosis of AOM. The effect of antibiotic use on loads, by ear state, was also investigated (data not included). No differences in the bacterial loads were observed, with or without antibiotic use, in swabs taken at a diagnosis of AOM or AOMwiP.

Viral-bacterial co-infections are often associated with antibiotic treatment failure [36]. In the Indigenous children in our study, antibiotic therapy had no discernable effect on bacterial loads. For densely colonised children with treatment failure, more work is needed to determine whether antiviral medication might be a valid adjunct to antibiotic treatment. Recently a randomized controlled trial in the United States found that children receiving oseltamivir (Tamiflu) treatment within 48 hours of clinic presentation for laboratory confirmed influenza were considerably less likely to progress to AOM. This effect was greatest for children between the ages 1-5 years old where the incidence of AOM was the greatest [37].

The limited clinical impact of PCV7 on OM in indigenous children is largely attributed to pneumococcal serotype replacement. This study however, suggests that the influence of respiratory viruses on OM in this population may be undervalued. A review by Cripps and Otczyk in 2006 promotes a vaccine that includes both bacterial and viral antigens [38] and others have shown that the influenza vaccine can reduce overall AOM incidence by greater than 30% [39-41]. Whether vaccines for individual viruses would have an impact on endemic OM is unclear, but protection against multiple pathogens may be needed to attenuate the complex interplay between viruses and bacteria in OM in high risk populations.

## Conclusions

For Indigenous children with OM this study highlights the frequency of viral infection, describes increasing bacterial density with progression to AOM, suggests a synergy of viral-bacterial co-infection, and implicates adenovirus in AOMwiP pathogenesis. Antibiotic and antiviral therapy is ever expanding and improving and it seems likely that only vaccines and/or chemotherapies targeting multiple bacterial and viral species, in combination with successful social interventions to reduce cross infection, will reduce OM prevalence, morbidity and sequelae in high risk populations. The introduction of the 10-valent pneumococcal conjugate vaccine, with broader pneumococcal serotype coverage and potential protection from the addition of the *H. influenzae *protein D antigen is possibly the first step in the right direction.

## Competing interests

The authors declare that they have no competing interests.

## Authors' contributions

MB performed the DNA extractions, bacterial loads PCR's, data analysis and interpretation of results. MB is the author of this work. AC contributed to the design of the larger case control study from which these samples were drawn, and assisted with statistical analysis and editing. HSV provided expertise both to the design of the RTQ-PCR and the writing of this paper. TS's laboratory screened the swabs for the 17 respiratory viruses and TS assisted with interpretation of the virus PCR results. MN provided clinical interpretation of the virology. DW performed the DNA extractions and PCR assays to identify the viruses. JM assisted with and proofed the statistical calculations. AJL concepted this study and contributed significantly to the analysis and editing of this manuscript. All authors have read and approved the final version.

## Pre-publication history

The pre-publication history for this paper can be accessed here:

http://www.biomedcentral.com/1471-2334/11/161/prepub
